# Contrast thresholds for detection of various iodine concentrations in subtraction CT and dual‐energy CT systems

**DOI:** 10.1002/acm2.13834

**Published:** 2022-11-05

**Authors:** Anahita Heshmat, Izabella Barreto, Lynn Rill, Sitong Liu, Romin Patel, Manuel Arreola

**Affiliations:** ^1^ Department of Radiology, College of Medicine University of Florida Gainesville Florida USA

**Keywords:** computed tomography, contrast thresholds, contrast‐to‐noise ratio, dual‐energy CT, iodine map, limit of blank, limit of detectability, minimum iodine concentration, Rose criteria, subtraction CT

## Abstract

**Objective:**

To estimate the minimum iodine concentrations detectable in simulated vessels of various diameters for both subtraction computed tomography (CT) and dual‐energy CT systems.

**Methods:**

Fillable tubes (diameters: 1, 3, and 5 mm) were filled with a variety of iodine concentrations (range: 0–20 mg/ml), placed in the center of 28‐mm cylindrical rods and surrounded with water. Rods with and without fillable tubes were placed in a 20‐cm cylindrical solid‐water phantom to simulate administration of iodine in blood vessels. The phantom was scanned with clinical subtraction CT (SCT) and dual‐energy CT (DECT) head protocols to assess the detection of minimum iodine concentrations in both systems. The SCT and DECT images were evaluated quantitatively with a MATLAB script to extract regions of interest (ROIs) of each simulated vessel. ROI measurements were used to calculate the limit of detectability (LOD) and signal‐to‐noise ratio of Rose criteria for the assessment of the contrast thresholds.

**Results:**

Both SNR_Rose_ and LOD methods agreed and determined the minimum detectable iodine concentration to be 0.4 mg/ml in the 5‐mm diameter vessel for SCT. However, the minimum detectable concentration in the 5‐mm vessel with DECT was 1 mg/ml. The 3‐mm vessel had a minimum detectable concentration of 0.8 mg/ml for SCT and 2 mg/ml for DECT. Lastly, the minimum detectable iodine concentration for the 1‐mm vessel was 10 mg/ml for SCT and 10 mg/ml for DECT.

**Conclusion:**

In this phantom study, SCT showed the capability to detect lower iodine concentrations compared to DECT. Contrast thresholds varied for vessels of different diameters and the smaller vessels required a higher iodine concentration for detection. Based on this knowledge, radiologists can modify their protocols to increase contrast enhancement.

## 1 INTRODUCTION

Evaluating the usage of contrast agents for different imaging examinations is an ongoing task.^1^, ^2^ Iodine has been the contrast agent of choice for computed tomography (CT) imaging applications in order to increase photoelectric absorption and enhance the visibility of internal anatomical structures such as vasculature.^3^ New innovative CT technologies such as dual‐energy CT (DECT) and subtraction CT (SCT) rely on accurate iodinated contrast use.^4^ Administration of too low or too high of contrast concentration can affect CT image quality through low contrast enhancement or beam hardening artifacts. The development of processing‐based technologies has expanded the availability of quantitative CT iodine map (IM) images in DECT and SCT. DECT IM images display the distribution of iodinated contrast agents in the body, offering local perfusion information and quantification of iodine, useful for lesion characterization.^5^ DECT can also produce virtual non‐contrast (VNC) images by removing iodine content from the data to simulate an unenhanced scan.^3^

Alternatively, SCT has the ability to remove bone, calcium, and stents from CT images, allowing clinicians an unobstructed view of iodine distribution in arteries.^6^ As SCT subtracts an unenhanced scan from a contrast‐enhanced scan, it can also generate IM images and may be an attractive alternative to DECT as SCT does not require additional hardware. Both DECT and SCT provide quantitative images, where the signal in the IM image is proportional to the iodine concentration.^7^

With growing interest in these new CT technologies, researchers have evaluated the informational accuracy of DECT and SCT imaging.^1^, ^7^ Although some preliminary studies have assessed simulations of DECT and comparison of DECT with SCT, to our knowledge, no studies have assessed iodine detection in the presence of clinically relevant anatomical materials, sizes or provided iodine threshold detection methods for scanners that do not provide iodine quantification. This study evaluates limits of iodine detection in both DECT and SCT systems by providing the contrast thresholds of minimum iodine concentrations detectable in a variety of sizes of simulated vessels in both systems.

## 2 MATERIALS AND METHODS

### 2.1 Phantom configurations

**FIGURE 1:**
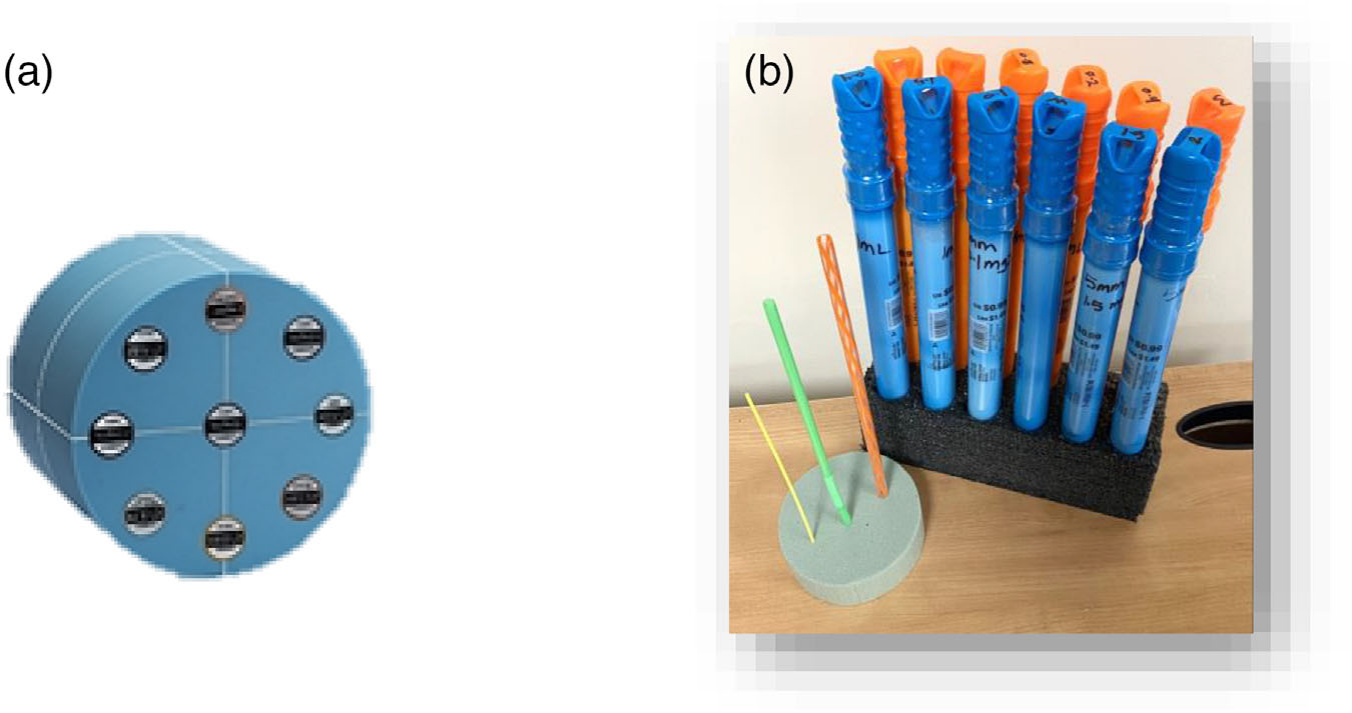
(a) A cylindrical Gammex phantom with two sizes representing an adult head and torso; (b) plastic rods filled the voids in the head phantom. These contained water and 1‐, 3‐, and 5‐mm diameter tubes to simulate different‐sized vessels.

A multi‐energy CT phantom (Gammex, Inc., Madison, WI) was used in this study.^8^ The solid‐water phantom is 16‐cm in length and has two sections: a 20‐cm insert that simulates an adult head, as shown in Figure 1a. The head phantom contains 10 28‐mm wide voids that can be filled with interchangeable rods of different materials. Although the vendor provides proprietary solid rods with the phantom, in this study, these rods were replaced with custom plastic rods, which were 40 cm in length and 28 mm in diameter (Figure 1b). The plastic rods had thin walls to minimize any effect on the CT number of the materials inside the rods in the phantom images. Furthermore, to simulate a variety of vessels, different smaller fillable tubes were chosen based on the smallest adult arteries (1, 3, and 5 mm) representing the bronchial, cerebral, and carotid arteries, respectively (Figure 1b).^9^ The tubes were filled with iodinated contrast material (iohexol, Omnipaque 350; GE Healthcare Ireland, Cork, Ireland) with various concentrations of iodine (0.1–20.0 mg/ml). Moreover, two different sets of iodine dilutions were created: one diluted with water (0.1, 0.2, 0.4, 0.8, 1, 1.5, 2, 5, 10, and 20 mg/ml) and the other diluted with bovine blood with the same concentration levels. Three different sets of each iodine concentrations were performed with three different chemists, following the same dilution method for accuracy of dilutions. Each set was scanned with the same CT protocol to measure CT numbers and further validated the accuracy of iodine concentrations. A *p*‐value of greater than 0.05 was indicative that there is no difference among the three sets of dilutions. Furthermore, the measured CT numbers of iodine concentrations were compared to the calculated CT numbers of the iodine concentrations with The National Institute of Standards and Technology (NIST) that validated the diluted iodine concentrations with water and blood. The bovine blood had a CT number of 44 HU, similar to human blood.^6^

The thin tubes, simulating vessels, were then placed in the center of the plastic rods and surrounded with water, and the rods were placed in the phantom to simulate a CT patient injected with iodine in arteries surrounded with soft tissue. The phantom was centered in the gantry's isocenter. The plastic rods were placed in the eight voids of the head phantom located at a radius of 7.5 cm from the center of the phantom to reduce the effects of cupping artifacts in subsequent measurements, which could alter the accuracy of the CT numbers of the iodine concentrations.

### 2.2 Scanner and reconstruction protocols

**FIGURE 2:**
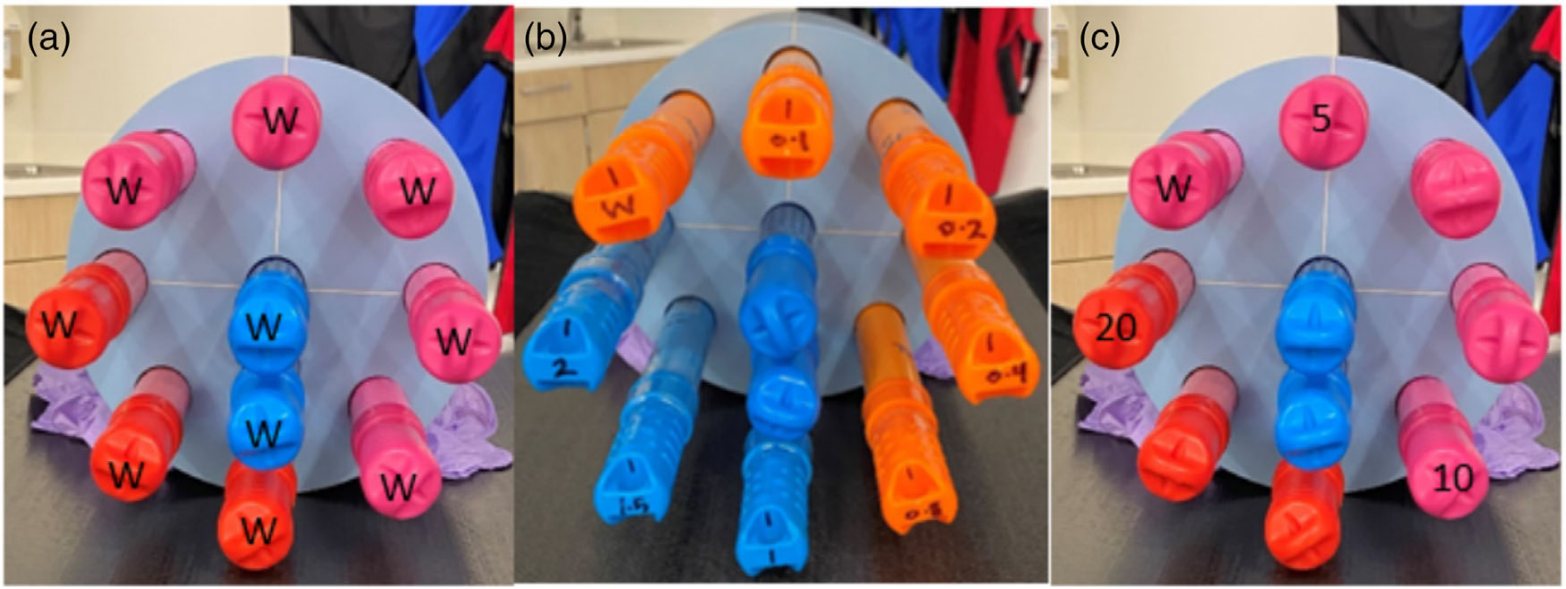
(a) Head phantom with water rods (W) to simulate a non‐contrast scan; (b) head phantom with the lower iodine concentrations (water‐2 mg/ml); (c) head phantom with the higher iodine concentrations (water, 5, 10, and 20 mg/ml)

The DECT and SCT images were acquired by a 320‐detector CT scanner (Canon Aquilion ONE/Genesis Edition, Canon Medical Systems USA, Inc., Tustin, CA). The phantom was scanned with the clinical head DECT protocol and the CTA head SCT protocol indicated in Table 1 with a fixed tube current. The phantom was scanned twice for each simulated vessel size, first with the low iodine concentrations (0.1–2 mg/ml of iodine), and again with the higher iodine concentrations (5, 10, and 20 mg/ml). The 11 o'clock rod in both scans contained a tube with pure water (W) to represent 0‐mg/ml iodine. The plastic rods were positioned and scanned as shown in Figure 2.

**TABLE 1:** Scan and reconstruction parameters for subtraction computed tomography (SCT) and dual‐energy computed tomography (DECT)

	SCT	DECT
Protocols	CTA head	Head
Tube voltage (kVp)	120	135 + 80
CTDI_vol_ (mGy)	53.0	54.6
DLP (mGy cm)	848	873.6
Tube current (mA)	400	100 + 570
Rotation time (s)	0.5	0.5
Field‐of‐view (mm) Bowtie filter size	220.3	220.3
Bowtie filter size	S	S
Reconstruction kernel (FC)	FC64	FC64/13
Iterative reconstruction method	AIDR‐3D (mild strength)	AIDR‐3D (mild strength)
Slice thickness (mm)	1, 3, and 5	1
Pitch	0.625	0.625
Field of view (mm^2^)	200 × 200	200 × 200
Detector configuration (mm^2^)	320 × 0.5	320 × 0.5

Two scans were acquired for the SCT protocol, as it requires pre‐ and post‐contrast images with a total radiation dose of 53 mGy. The head phantom with all water rods (W) was scanned for the initial non‐contrast image to simulate when the patient is scanned without any iodine contrast as shown in Figure 2a. The contrast image was acquired with rods containing the tubes of various iodine concentrations as shown in Figure 2b,c to simulate the scan with administration of iodine in the patient. All DECT scans were acquired with the head phantom containing iodine tubes, acquiring both 80‐ and 135‐kVp images.

**FIGURE 3:**
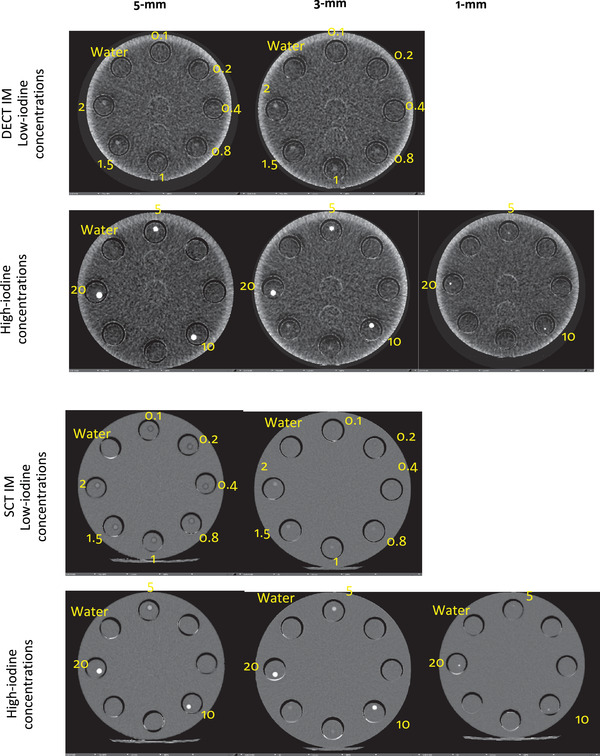
Dual‐energy computed tomography (DECT) and subtraction computed tomography (SCT) images of iodine map (IM) in variety of tube diameters (5, 3, and 1 mm) and iodine concentrations in mg/ml (overlaid in yellow text on the images)

Once all the scans were completed (Figure 3), images were reconstructed with different slice thicknesses (1, 3, and 5 mm). DECT's virtual monochromatic image, VNC, and IM as well as SCT's IM images were created on the scanner console according to vendor recommendations. All images were evaluated with a MATLAB script (The MathWorks, Inc, Natick, Ma [MATLAB R2019a]) to automatically place regions of interest (ROIs) in the image location of the tubes inside the plastic rods. The mean CT numbers, standard deviations, and the area of the ROI in a number of pixels were collected for each simulated vessel by ROI analysis.

### 2.3 Detection methods

To define vessel detectability and calculate the contrast thresholds of iodine concentrations in both DECT and SCT systems, two different detection methods were utilized. First, Rose criteria were calculated based on signal‐to‐noise ratio (SNR) and test patterns for reliable detection of objects.^10^ The detection limit of an imaging system depends on lesion to background contrast and the noise in the background, which is contrast‐to‐noise ratio (CNR). For tomographic imaging systems, the SNR_Rose_ is expressed as the lesion CNR multiplied by the square root area of the object and the standard deviation of the background as shown in the following equation^10^:

(1) $$\;\mathrm{SN}R_{\mathrm{Rose}}=\;\mathrm{CNR}\times \sqrt{A\times ø}$$

If the SNR_Rose_ is greater than 5.0, the object is considered detectable.^10^

The second detection method that was used, limit of detectability (LOD), was calculated based on detection as defined by the Clinical and Laboratory Standards Institute (CLSI) and the International Union of Pure and Applied Chemistry (IUPAC). The two sensitivity metrics include the limit of blank (LOB) and the LOD, both with units of HU. LOB is the highest apparent analyte concentration measured for a blank sample (water/blood) containing no analyte (i.e., mean CT number of a blank sample, mean_blank_). A 5% false‐positive detection rate (1.645 multiplicative factor) and noise (SD_blank_) of the blank sample are accounted for as shown in the following equation^11^:

(2) $$\mathrm{LOB}\left(\mathrm{HU}\right)\;=\mathrm{mea}n_{\mathrm{blank}}\;+1.645\times SD_{\mathrm{blank}}$$

LOD is the lowest analyte concentration to be differentiated from the LOB. This also accounts for a 5% false‐positive detection rate and noise of the low concentration sample as shown in the following equation^11^:

(3) $$\mathrm{LO}D_{\mathrm{signal}}\left(\mathrm{HU}\right)\;=\;\mathrm{LOB}+1.645\times SD_{\mathrm{low}}$$

In order to detect the minimal iodine concentrations in different sizes of simulated vessels, water and blood were each utilized as an LOB. All simulated vessels containing iodine were expected to have a CT number higher than the CT number of water to show that they were enhancing.

A CT‐number‐to‐iodine‐concentration conversation factor was calculated to allow a user to convert a measured CT number in the IM to an iodine concentration. A linear regression analysis of the known iodine concentration as a function of measured CT number resulted in the slope and *y*‐axis intercepts for the conversion factor. Additionally, correlation coefficients between the calculated and known iodine concentrations were calculated for the data. The *C*_slope_, with units of mg/ml/HU, and *C*_int_, with units of mg/ml, was used to convert a measured CT number to an iodine concentration as shown in the following equation:

(4) $$\begin{array}{c} C\;\;\left(\mathrm{mg}/\mathrm{ml}\right)\;=\;C_{\mathrm{slope}}\left(\frac{\frac{\mathrm{mg}}{\mathrm{ml}}}{\mathrm{HU}}\right)\;\times \mathrm{CT}\;\left(\mathrm{HU}\right)+C_{\mathrm{int}}\left(\frac{\mathrm{mg}}{\mathrm{ml}}\right) \end{array}$$

where the fitting parameters of this equation are CT scanner dependent.

Blood was used as an LOB for differentiating blood–iodine mixtures in DECT, whereas water was used as an LOB for all other evaluations. SCT and DECT images of the phantom containing simulated vessels were evaluated with a MATLAB script that calculated the CNR, SNR_Rose_, LOB (HU), LOD (HU), as well as LOD (mg/ml) to calculate contrast thresholds.

## 3 RESULTS

**FIGURE 4:**
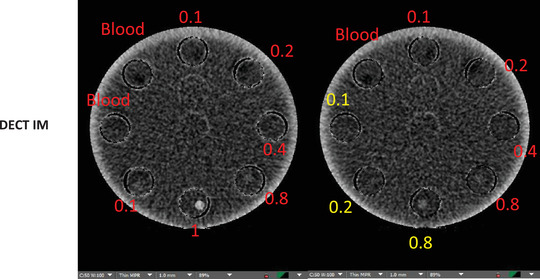
Dual‐energy computed tomography (DECT) iodine map (IM) images differentiating pure blood from a variety of iodine concentrations (0.1, 0.2, 0.4, 0.8, and 1 mg/ml) of blood–iodine mixtures indicated in red. Water–iodine mixtures (0.1, 0.2, and 0.8 mg/ml) are indicated in yellow.

**FIGURE 5:**
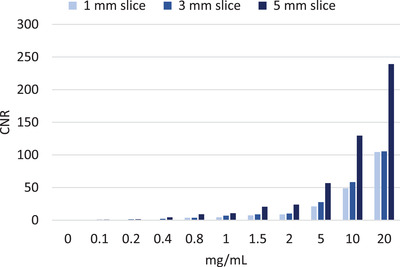
Bar graphs show contrast‐to‐noise ratio (CNR) of subtraction computed tomography (SCT) iodine map (IM) reconstructed with different slice thicknesses for all iodine concentrations.

Table 2 shows the calculated SNR_Rose,_ CNR, minimum iodine concentrations detected, and the corresponding detectable iodine concentrations in all tube diameters for both SCT and DECT. Both limits of detectability and SNR_Rose_ methods agreed, determining that the minimum detectable iodine concentration in the 5‐mm tube was of 0.4 mg/ml for SCT and 1.0 mg/ml for DECT. The 3‐mm tube had a minimum detectable concentration of 0.8 mg/ml for SCT and 2 mg/ml for DECT. The minimum detectable iodine concentration for the 1‐mm tube was 10 mg/ml for SCT and 10 mg/ml for DECT. Table 2 also indicates that the corresponding detectible iodine concentrations have higher concentration compared to the calculated LOD_signal_ in mg/ml. In Figure 4, it is evident that DECT's IM image is completely subtracting blood from the blood‐iodine mixtures to display iodine distribution for different concentrations. As blood is denser than water in Figure 4b, it is apparent that water–iodine mixtures have higher CT values than the iodine concentrations diluted with blood. This has to do with the water and blood CT numbers getting subtracted from iodine to only show iodine distribution. Table 3 shows the calculated SNR_Rose_, CNR, and minimum iodine concentrations detected in DECT for iodine concentrations diluted with blood. Both limits of detectability and SNR_Rose_ methods agreed, determining the minimum detectable iodine concentration of 0.8 mg/ml with a CNR of 3.2. Furthermore, technical parameters had a great impact on CNR. The CNRs of iodine concentrations with various slice thicknesses are shown in Figure 5 and Table 4. It is evident that with thicker slice thickness of 3‐ and 5‐mm, lower iodine concentrations are detectable as compared to the 1‐mm slice thickness.

**TABLE 2:** Detectability of minimum iodine concentration calculated with limit of detectability and SNR_Rose_ criteria

	Tube diameter	LOB_signal_	LOD_signal_		Corresponding detectable iodine concentration (mg/ml)	SNR_Rose_	CNR	Corresponding detectable iodine concentration (mg/ml)
Systems	(mm)	(HU)	(HU)	(mg/ml)				
	5	4.93 ± 0.05	9.87	0.32 ± 0.02	0.4	6.27 ± 0.11	0.60	0.4
SCT	3	7.36 ± 0.03	14.73	0.67 ± 0.09	0.8	8.44 ± 0.57	3.79	0.8
	1	6.98 ± 0.09	45.98	7.33 ± 0.13	10.0	29.05 ± 0.87	48.18	10.0
	5	23.21 ± 0.10	26.90	0.87 ± 0.72	1.0	6.23 ± 0.88	1.20	1.0
DECT	3	41.21 ± 1.90	50.15	1.76 ± 0.80	2.0	6.57 ± 0.98	3.48	2.0
	1	43.29 ± 1.70	63.05	10.00 ± 1.20	10	8.77 ± 1.3	4.38	10

*Note*: Concentrations are presented as mean ± standard deviation.

Abbreviations: CNR, contrast‐to‐noise ratio; DECT, dual‐energy computed tomography; SCT, subtraction computed tomography.

**TABLE 3:** Dual‐energy computed tomography (DECT)’s iodine map (IM) image detecting iodine from blood–iodine mixtures

			**LOD_signal_**		**Corresponding iodine concentration (mg/ml)**			**Corresponding iodine concentration (mg/ml)**
**Systems**	**Tube diameter (mm)**	**LOB_signal_ (HU)**	**(HU)**	**(mg/ml)**	**SNR_Rose_**	**CNR**	
DECT	5	16.39 ± 0.15	68.20	0.78 ± 0.05	0.8	16.64 ± 1.9	3.2	0.8

Abbreviation: CNR, contrast‐to‐noise ratio.

**TABLE 4:** Contrast‐to‐noise ratio (CNR) of iodine concentrations with variety of slice thickness for subtraction computed tomography (SCT)

Iodine concentrations (mg/ml)	1 mm CNR	3 mm CNR	5 mm CNR	SD
0	0	0	0	0
0.1	0.06	0.91	1.02	0.42
0.2	0.07	1.33	1.50	0.63
0.4	0.60	2.11	4.47	1.59
0.8	3.79	3.79	9.08	2.49
1	4.43	7.01	10.74	2.58
1.5	7.42	8.97	20.63	5.89
2	8.87	10.22	23.90	6.78
5	21.20	27.71	56.88	15.51
10	48.81	58.33	129.58	36.04
20	104.49	105.51	239.22	63.27

## 4 DISCUSSION

In this study, we found that SCT had superior detectability and CNR compared to DECT. Moreover, discrimination of details smaller than 5 mm was possible at lower iodine concentrations in SCT as compared to DECT.

Postprocessing algorithms have introduced DECT into the stroke imaging routine, where DECT's three‐material decomposition algorithm can be used to differentiate residual iodinated contrast staining from hemorrhagic transformations in follow‐up stroke patients through the use of IM and VNC images. IM images enhance residual iodine contrast, if present, and suppress other tissues, such as hemorrhagic blood. VNC images suppress residual iodine and maintain other tissues, allowing for the improved visualization of hemorrhagic blood.^12^

An advantage of SCT over DECT is the signal difference between pre‐ and post‐contrast acquisitions is larger than the signal associated with the iodine attenuation difference at two tube voltages, as used in DECT.^7^ From the detectability data, it is evident that SCT can be obtained at lower dose level and have the same results as DECT. However, SCT is only useful for clinical applications that utilize administered contrast agents, and not useful for differentiating materials in the body, such as bone, calcifications, and blood.^13^

As the scanner we investigated in this study does not provide iodine concentration (mg/ml), but rather CT numbers (HU), it was vital to create a conversion factor that allows the user to convert a CT number to a concentration of iodine in the IM. Potential uses of the conversion factor may include iodine uptake measurements in clinical patient data for quantitative imaging. Assessment of the quantitative data using the Rose criteria and LOD methods could aid researchers to quantify limiting iodine concentrations in mg/ml for scanners that do not have the capability of displaying quantification of iodine. This will guide radiologists in case they need to increase the contrast dose for detection of smaller vessels.

Previous studies had scanned a variety of iodine concentrations and rod vessel diameters and found SCT resulted in higher CNR compared to DECT.^7^ However, they used a dual‐source CT system, which is limited to material decomposition in the image domain, as well as prone to detecting more cross‐scattered radiation. Furthermore, the authors compared a Siemens DECT system with a Canon SCT system, using two different iterative reconstruction algorithms. They also did not evaluate iodine discrimination for image thicknesses other than 1 mm. One group identified minimum detectable iodine concentrations can range between 0.02 and 0.5 mg/ml, but noted these results are highly dependent on the DECT manufacturer and on the beam's spectral separation.^14^ The study did not evaluate a Canon DECT system and assessed detectability of iodine relative to water, rather than more clinically‐relevant tissues such as acute hemorrhagic blood.

Our study had limitations. The Rose criteria method was based on SNR_Rose_ exceeding five to define the iodine concentrations detectable; however, the minimum detectable iodine concentration had a higher SNR_Rose_ than 5, which means we should have been able to detect a lower concentration than the one we examined. The LOD method depended on the standard deviation of the mean signal in the blank rod, which ideally should be performed with more than 20 repetitions of measurements to ensure accuracy; however, for this study, only 10 repetition measurements were performed. Furthermore, our study was a phantom study and even though we mimicked many tissue‐equivalent materials and sizes to a patient scenario; however, these data were not confirmed with a clinical patient experiment to take into consideration different adult head thicknesses and the image noise from a real skull. A future study will compare this phantom study to an actual clinical patient study where patient's various thicknesses will be evaluated as well as the image noise of a real skull.

In conclusion, SCT showed the capability to detect lower iodine concentrations compared to DECT. Contrast thresholds varied for vessels of different diameters and the smaller vessels required a higher iodine concentration for detection. Based on this knowledge, radiologists can modify their protocols to increase contrast enhancement in images by either selecting alternative CT technologies or by increasing iodine enhancement for small vasculature.

## AUTHOR CONTRIBUTIONS

Anahita Heshmat conceived, planned, and carried out the experiments. Anahita Heshmat and Romin Patel carried out the simulations. Sitong Liu contributed sample preparation. Anahita Heshmat, Izabella Barreto, Lynn Rill, and Manuel Arreola contributed to the interpretation of the results. Anahita Heshmat took the lead in writing the manuscript. All authors provided critical feedback and helped shape the research, analysis and manuscript.
